# Annotations

**Published:** 1906-09-29

**Authors:** 


### ANNOTATIONS.

### The Hampstead General Hospital and Out-Patients.

The Hampstead General Hospital Council received a deputation from the medical practitioners resident in the district desirous of pointing out certain defects in the management of the hospital, in the hope that they might be remedied in an early stage, and that the harm caused by ill-regulated out-patient departments in other hospitals thereby avoided. Dr. Ford Anderson moved a resolution to the effect that the out-patient department in Hampstead Hospital was unnecessary and exercised a demoralising effect upon the inhabitants. Despite the enormous sums contributed by the public, the hospitals were always crying out for more aid, largely owing to the expensive bills incurred by their bloated out-patient departments. One crux of the question lies in the undefined and almost undefinable "too poor." There is no need to labour the difficulty of fixing a standard of wealth or poverty, for these are relative matters, relative on the one hand to the domestic calls upon income, and upon the other to the protraction and nature of illness. It is platitudinous to observe that the clerk with a large family may be far poorer on fifty shillings a week than the unmarried labourer on twenty-two; but the point is vital to a proper estimate of the position. The wide scope of the problem does not admit of more than a general assumption as to the abuse of hospitals by those who can afford to pay. While gross cases of abuse are rare, so long as the drink and tobacco bills of the nation remain what they are it is impossible to resist the conclusion that the publican and tobacconist grow rich at the expense of the public charities and of the private doctor.

### Reported Cure of Sleeping Sickness.

Recently reports, emanating from Brussels, have appeared in the lay press as to the apparent cure of cases of sleeping-sickness. It has been stated that three cases of trypanosomasis (sleeping-sickness) have been cured at the Bacteriological Institute of Leopoldville, and that two Europeans received some weeks ago into a sanatorium at Watermael, near Brussels, are now pronounced to be cured, although the disease had reached an advanced stage, the treatment employed being based on the simultaneous use of atoxyl and strychnine. Such statements must be received with great caution. It is true that animals suffering from trypanosomiasis have apparently been cured—at least all trypanosomes have disappeared from their blood for varying periods of time—by treatment with arsenic, atoxyl, and various anilin preparations; but the question as to how similar lines of treatment will affect human beings is still in its infancy. If we consider trypanosomiasis as the early stage of sleeping-sickness then there appears to be some hope, for one case is reported to be well and healthy, now five years after trypanosomes were demonstrated in the blood. Still, though granting this, one cannot put any faith in the recent reported cures from Brussels until two, three, or more years have elapsed, because it is quite possible that the disease is only temporarily in abeyance and may break out again at any time within those limits. Cases have been known to develop sleeping-sickness years after they have left the endemic area, and this simply means they have been harbouring the parasite in their bloods all the time without symptoms. As regards cases with the definite symptoms of sleeping-sickness well developed, or which have reached an advanced stage, as the reports term it, all past experience tends to show that they are uniformly fatal. Such being the case, it will be specially interesting to follow the subsequent histories of the cases under review. As the matter is one of so much importance, it is to be sincerely hoped they will not be lost sight of, and everyone will look forward in the course of the next two or three years to hear further particulars as to the permanency or otherwise of the cure.

### Flock Mattresses.

Many sanitarians have tried to draw attention to the dangers which may exist in the wool mattresses so commonly used by the poorer classes, and often to be found, at least in the servants' quarters, in the houses of those who are not poor. The wool mattress is cheap and, at least at first, comfortable. After not very prolonged use it tends to become hard and lumpy, as the fragments of wool and cloth which form the stuffing begin to stick together. In other countries where wool mattresses are used it is customary to have them opened and cleaned every year, but in England the matted flocks are allowed to remain until the outer covering wears into holes. Even if the flocks were originally perfectly clean, the condition of the mattress at the end of some years must be far from sanitary; and in the cheapest qualities the filling is vile in itself—scraps of cloth too worthless to be worked up again into shoddy, simply torn up into small fragments and stuffed into the mattress without cleansing. They may be infected when they are first used, and in any case they form a good breeding-ground for bacteria. Dr. Leo Taylor, the analyst to the Hackney Borough Council, has recently been making some investigations, and he declares that even after they have been washed materials of this class—the better qualities, too—are capable of yielding 7,500,000 colonies of bacteria per gramme of flock. This is not comfortable news; and if this be the case with the best washed flock, what may one imagine of inferior and unwashed qualities! In a private house a bed may be in use for years without being an inevitable danger to health, but as we often find these mattresses in seaside and country lodgings, where the occupants of the bed change from week to week, the possibilities of infection lying *perdu* in the mattress can hardly be ignored. That the spread of disease has ever been traced to such a source we do not know, but there is little doubt of the possibility of it, and it might be kept in mind when mysterious illnesses arise without an obvious cause.

